# Supplementary material for: Mutations in PCYT2 disrupt etherlipid biosynthesis and cause a complex hereditary spastic paraplegia
Source: Brain. 2019 Oct 22;142(11):3382–97. doi: 10.1093/brain/awz291 (PMC6821184; doi:10.1093/brain/awz291)
Supplement: awz291_Supplementary_Data [file awz291_supplementary_data.zip › awz291-Suppl_data/Supplementary_Data4.zip!/Supplementary information 3 - fibroblast lipidomics.zip!/Fibroblast lipidomics/Controle vs Patient1/Statistical plots and data/PE species/Boxplots.pdf]

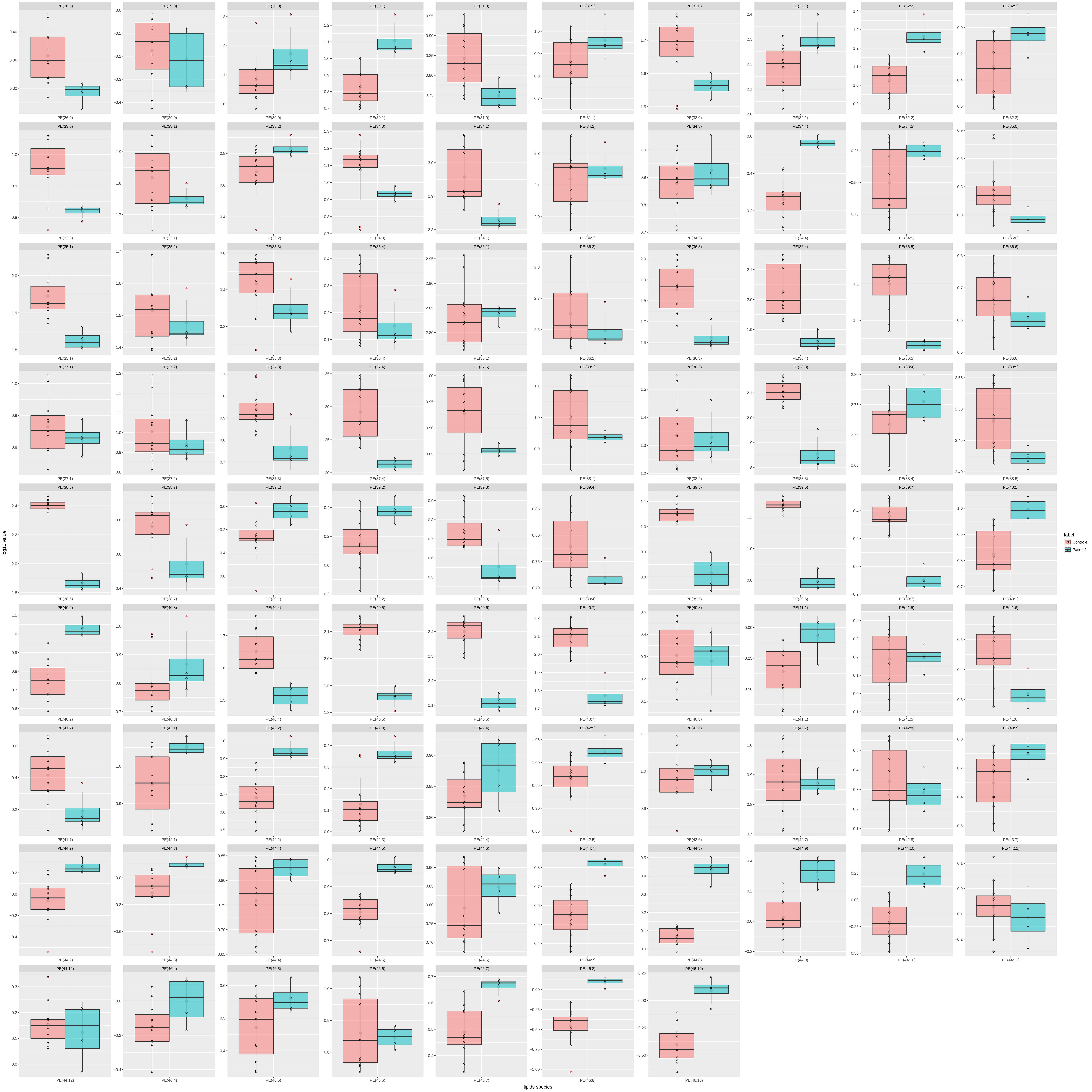
PE(26:0)
PE(29:0)
PE(30:0)
PE(30:1)
PE(31:0)
PE(31:1)
PE(32:0)
PE(32:1)
PE(32:2)
PE(32:3)
PE(33:0)
PE(33:1)
PE(33:2)
PE(34:0)
PE(34:1)
PE(34:2)
PE(34:3)
PE(34:4)
PE(34:5)
PE(35:0)
PE(35:1)
PE(35:2)
PE(35:3)
PE(35:4)
PE(36:1)
PE(36:2)
PE(36:3)
PE(36:4)
PE(36:5)
PE(36:6)
PE(37:1)
PE(37:2)
PE(37:3)
PE(37:4)
PE(37:5)
PE(38:1)
PE(38:2)
PE(38:3)
PE(38:4)
PE(38:5)
PE(38:6)
PE(38:7)
PE(39:1)
PE(39:2)
PE(39:3)
PE(39:4)
PE(39:5)
PE(39:6)
PE(39:7)
PE(40:1)
PE(40:2)
PE(40:3)
PE(40:4)
PE(40:5)
PE(40:6)
PE(40:7)
PE(40:8)
PE(41:1)
PE(41:5)
PE(41:6)
PE(41:7)
PE(42:1)
PE(42:2)
PE(42:3)
PE(42:4)
PE(42:5)
PE(42:6)
PE(42:7)
PE(42:8)
PE(43:7)
PE(44:2)
PE(44:3)
PE(44:4)
PE(44:5)
PE(44:6)
PE(44:7)
PE(44:8)
PE(44:9)
PE(44:10)
PE(44:11)
PE(44:12)
PE(46:4)
PE(46:5)
PE(46:6)
PE(46:7)
PE(46:8)
PE(46:10)
log10 value
lipids species
label
Controle
Patient1
